# Supplementary figures and images for: Cell type mapping of mild malformations of cortical development with oligodendroglial hyperplasia in epilepsy using single‐nucleus multiomics
Source: Epilepsia. 2025 Apr 28;66(8):3064–80. doi: 10.1111/epi.18413 (PMC12371647; doi:10.1111/epi.18413)

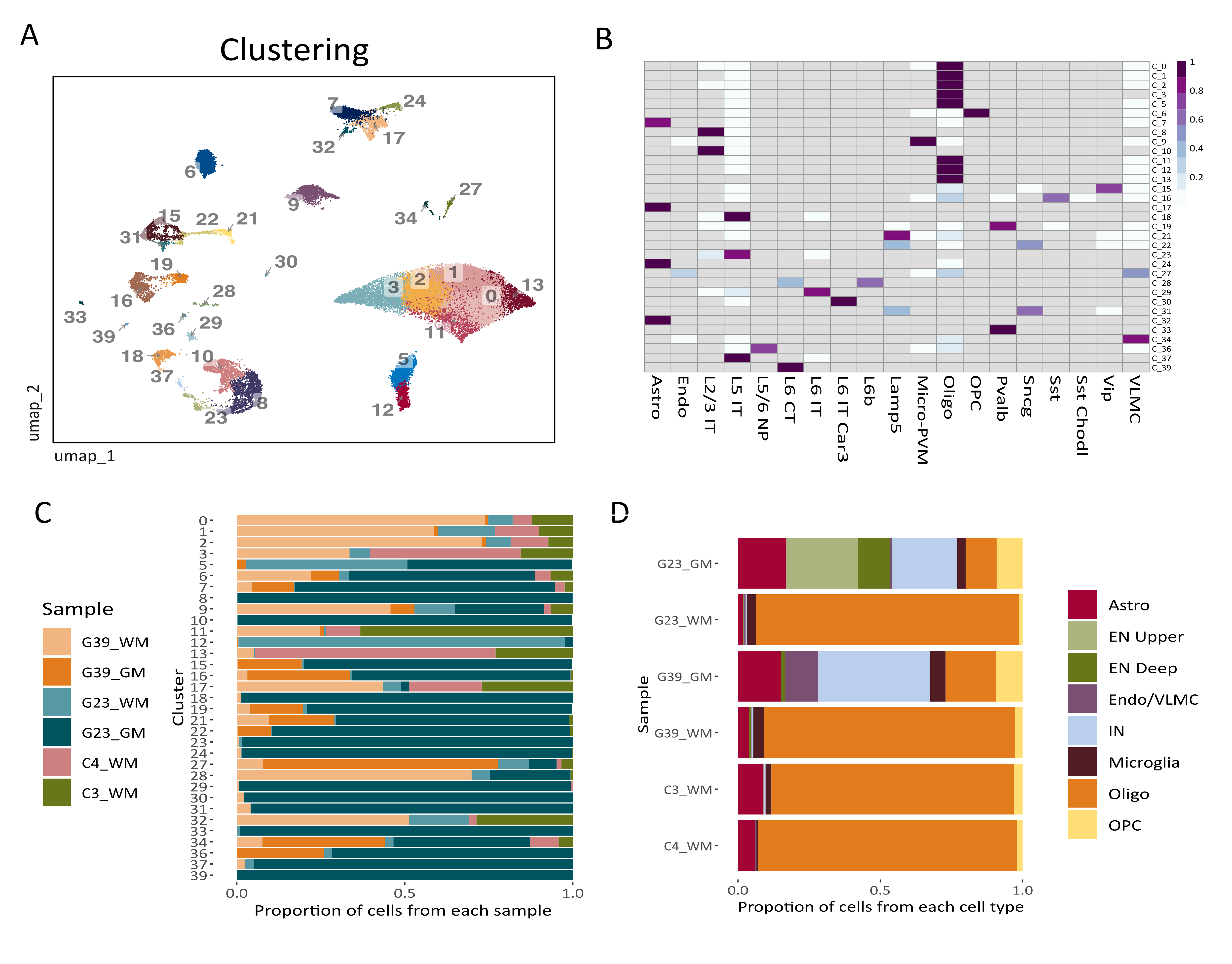

Supplement: Supplementary file 1 — Figure S1. [file EPI-66-3064-s004.tif]

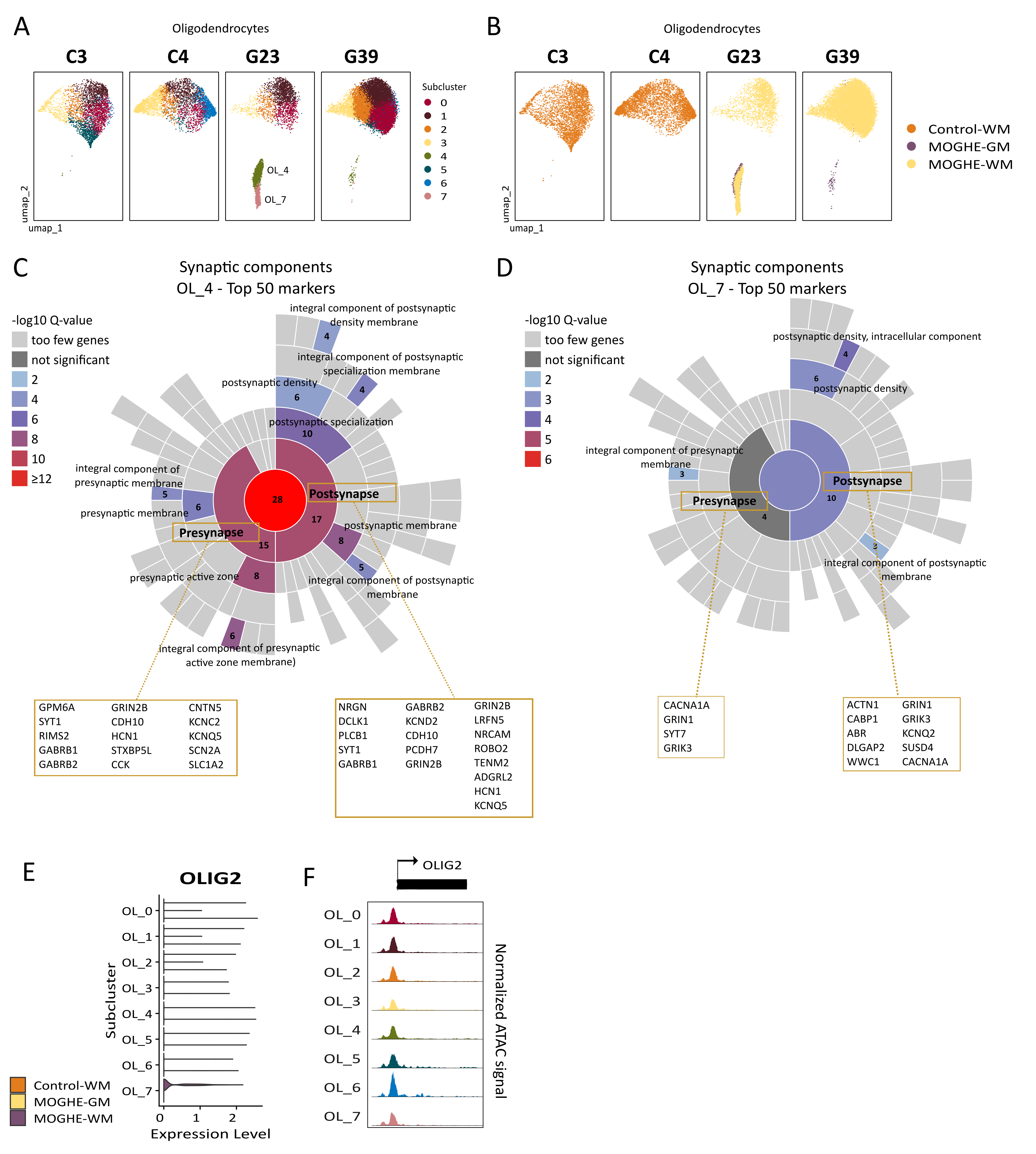

Supplement: Supplementary file 2 — Figure S2. [file EPI-66-3064-s005.tif]
